# Remote Photoplethysmography Using Triple-Head Spatio-Temporal Transformer with Reaction-Driven Gating and Illumination Separation

**DOI:** 10.3390/s26113490

**Published:** 2026-06-01

**Authors:** Ahmed Mehrez, Abdelwahab Alsammak, Shady Y. El-Mashad

**Affiliations:** Department of Electrical Engineering, Faculty of Engineering at Shoubra, Benha University, Cairo 11614, Egypt; asammak@feng.bu.edu.eg (A.A.); shady.elmashad@feng.bu.edu.eg (S.Y.E.-M.)

**Keywords:** rPPG, transformer, heart rate, motion artifacts, illumination noise

## Abstract

Remote Photoplethysmography (rPPG) provides a non-contact alternative to traditional heart rate monitoring. Estimating physiological signals from facial videos has recently attracted significant research interest. However, rPPG performance is sensitive to illumination variation and environmental interference, which can distort the extracted physiological signal. Since the background and face are affected by similar conditions, the effect of these conditions can be extracted from the background and isolated from the result. This paper proposes the Triple-Head Spatio-Temporal Transformer (TH-STT). TH-STT is a multi-task architecture designed to separate rPPG signals from environmental interference. In addition to facial tokens, a background anchor token is used as an environmental reference. Facial tokens and background anchor are processed using a shared transformer backbone. The proposed architecture has two auxiliary tasks to help purify the resulting rPPG. The Reaction-Driven Gating (RDG) mechanism was introduced, which tracks facial muscular activity. Furthermore, a Dynamic Anchor Locking (DAL) strategy is proposed to cancel environmental illumination interference. Experimental results on three benchmark datasets demonstrate improved and stable performance, with the TH-STT achieving a Mean Absolute Error (MAE) of 0.42 bpm on UBFC-rPPG and 1.08 on COHFACE.

## 1. Introduction

Several essential vital signs are monitored by healthcare providers. Heart rate provides the most vital information about a patient’s health. Heart rate measurement is used in many applications, such as medical and sports monitoring. Traditional methods for monitoring Heart Rate (HR), like electrocardiography (ECG) and photoplethysmography (PPG), use contact sensors and electrodes. These contact-based methods provide high-quality physiological measurements and can operate continuously for extended periods. However, their accuracy is heavily dependent on the physical interface, if a smart-watch is not positioned perfectly on the wrist, it can result in poor sensor contact that can lead to inaccurate results. They need continuous skin contact, which can be a challenge in some cases. Contact sensors may cause irritation, discomfort, and restrict movement. In such cases, a contactless method is highly desirable [1,2].

With the advancement of computer vision, recent research has proposed remote photoplethysmography (rPPG) to perform the measurement process remotely. rPPG relies on fine changes in skin color caused by blood flow, which are not visible to the naked eye [3]. These variations can be captured by RGB video cameras and processed to extract the blood volume pulse (BVP) signal. Accordingly, rPPG provides the ability to estimate physiological signals without direct contact. Therefore, it can be used in various applications, including telemedicine, driver monitoring systems, security and anti-spoofing, and human–computer interaction environments [4]. Remote photoplethysmography provides additional monitoring flexibility by eliminating physical sensor constraints. Recent advances in deep learning and mobile computing have accelerated the usage of rPPG models on mobile devices. rPPG frameworks will be used to allow high-accuracy monitoring directly on mobile phones for the general population.

Estimating rPPG accurately in real-world settings remains an open challenge. The main challenges are the change in ambient lighting conditions, facial movements, and selecting the optimal facial area to detect the pulse. Illumination variations affect the light reflected from the skin, thereby distorting the subtle color used for physiological signal estimation [5]. Additionally, motion resulting from head movements and facial expressions can produce noise that distorts the extracted signal [6]. Furthermore, the presence of external noise sources degrades the signal-to-noise ratio of the resulting pulse waveform. Practically, these noise frequencies often overlap with the frequency range of pulse signals. So, separation of these noise sources is fundamental for accurate HR estimation [7]. To solve these issues, recent studies have explored traditional signal processing and deep learning approaches. These studies aim to improve physiological signal extraction by removing environmental noise.

In addition to environmental noise, rPPG signals can also interfere with other physiological signals. Respiratory rate can interfere with the low-frequency pulse signal [8]. Moreover, emotional reactions change the facial geometry through time. Changes in face geometry can affect the skin-color reflections and, consequently, the resulting signal quality [9].

Recently, deep learning architectures have been used to extract physiological signals under challenging conditions. Spatio-temporal models, including convolutional neural networks (CNNs) and transformer-based architectures, have achieved good performance in estimating physiological signals [10,11]. Transformer models use attention mechanisms to capture long-range dependencies.

Final estimation accuracy depends strongly on post-processing, which acts as the critical bridge between the raw model output and the relevant heart rate estimate. A band-pass filter is applied to isolate the periodic Blood Volume Pulse from a spectrum by high-frequency sensor noise and low-frequency motion artifacts. However, selecting an optimal frequency band is non-trivial. A restrictive filter may clip main physiological variations, while an overly broad filter allows environmental noise to corrupt the signal. Consequently, the development of an intelligent post-processing stage that can adapt to the subject’s activity is as important as the primary feature extraction architecture itself.

Despite significant advances in deep-learning and transformer-based rPPG methods, three fundamental limitations still exist in current architectures. First, existing models use dense spatio-temporal tokenization that treats all facial regions with equal importance. This ignores the anatomical variability of facial regions. The forehead and cheeks provide purer physiological signals than the nose, periocular, and perioral regions, which may be corrupted by frequent micro-movements. Second, current architectures lack explicit mechanisms to disentangle environmental interference. They treat the transformer backbone as a black box for signal purification. Third, the tight coupling between deep feature extraction and physiological post-processing remains underexplored. Most methods apply static, predetermined frequency band-pass filters, which depend on the dataset-specific ground-truth statistics. While some models use a dataset-specific band adaptation, these approaches have a limited ability to generalize to dynamic scenarios where facial expressions and facial movements create artifacts that overlap with pulse frequencies.

These gaps motivate our proposed model, which introduces region-adaptive weighting, explicit noise disentanglement, and adaptive frequency post-processing. To achieve this, we propose a Triple-Head Spatio-Temporal Transformer (TH-STT) with three key innovations:

- We introduce Reaction-Driven Gating (RDG), a dynamic spatial masking mechanism guided by facial blendshapes.
- We propose Dynamic Anchor Locking (DAL) to estimate illumination noise using a background reference token.
- We design an Adaptive Frequency Window (AFW) to dynamically adjust bandpass filtering based on facial activity.

## 2. Related Work

This section shows the main research directions in rPPG. Starting with traditional signal-processing, deep learning–based, and recent transformer-based architectures designed for physiological signal estimation.

### 2.1. Traditional rPPG Signal Processing Methods

Early studies on rPPG focused on using traditional signal processing techniques. These studies have shown that blood pulse can be captured from facial videos under suitable illumination conditions [4]. Subsequent research was proposed to improve the accuracy of results against noise. The CHROM method enhances pulse signals by transforming RGB channels into a chrominance channel to reduce the effect of illumination variations [12]. CHROM’s fixed linear transformation assumes a stable skin reflection model that cannot adapt to variable illumination spectra or non-stationary motion patterns. This explains its performance degradation under dynamic conditions (e.g., changing light sources or subject movement), because the chrominance projection does not separate motion-induced color changes from blood volume variations. Later, the Plane-Orthogonal-to-Skin (POS) method improved signal extraction by using skin reflection properties [13]. POS projects color signals onto a plane orthogonal to the skin tone, which improves motion robustness over CHROM but still depends on a static model of skin reflectance. The method cannot learn task-specific features and has a limited accuracy when motion patterns (e.g., rapid rotation) or lighting spectra (e.g., LED versus fluorescent) deviate from the assumed model, leading to the high MAE values seen in Table 1 for challenging datasets.

These methods achieved a good performance under specific controlled environments. However, their dependence on predefined signal transformations limits their ability to work in real-world environments. Such models are still limited in handling real-world variations in lighting and motion using only fixed signal processing models.

### 2.2. Deep Learning–Based rPPG Methods

To overcome the limitations of traditional approaches, recent research used deep learning techniques that are capable of learning features from facial video. Convolutional neural networks (CNNs) have achieved a strong performance in modeling physiological signals. Several CNN-based architectures have been proposed to extract pulse signals from spatio-temporal representations of facial videos. Deep learning models such as DeepPhys [14] and PhysNet [6] demonstrated that convolutional networks can effectively capture color variations associated with BVP signals while learning motion mechanisms. These approaches improved estimation accuracy under moderate motion and illumination changes. However, DeepPhys’s 2D-CNN architecture processes normalized frame differences and has a limited temporal receptive field. This limited temporal field prevents the model from capturing complete cardiac cycles, making frequency-domain heart rate estimation unstable. Additionally, its motion branch cannot distinguish between rigid face motion and subtle skin color changes, as both appear as intensity variations to the convolutional kernels. PhysNet models temporal context by splitting long video sequences into several fixed-length temporal windows (typically 64 or 128 frames). For each individual window, it extracts localized spatio-temporal phase changes using stacked 3D convolutional layers and temporal max-pooling. However, PhysNet uses a heavy computational cost to deal with long temporal representations. TS-CAN [15] employs temporal shift modules to reduce computational cost, but the shift operations assume local temporal continuity, which degrades its performance under abrupt motion or illumination changes. Furthermore, the architecture lacks any explicit mechanism to separate physiological signals from environmental interference, relying entirely on data-driven learning.

CNN-based architectures remain limited in capturing long-term temporal dependencies within physiological signals. CNNs rely on local receptive fields. Therefore, modeling a signal over a longer duration may require more complex architectures. Consequently, research is moving towards transformer-based architectures, which are capable of capturing temporal signals in long video sequences.

### 2.3. Transformer-Based rPPG Architectures

Transformer architectures effectively model long-range temporal dependencies using self-attention mechanisms. The success of transformers in computer vision applications led to the development of Vision Transformers (ViT) [11]. These models have achieved strong performance in video tasks due to their ability to model spatial and temporal relationships. Therefore, transformer-based architectures have evolved to improve the rPPG signal extraction accuracy. PhysFormer [16] introduces a temporal difference transformer architecture. The model aggregates global spatio-temporal features using self-attention mechanisms. An extension of this work, PhysFormer++ combines a Slow-Fast architecture designed to capture both coarse and fine temporal information in video sequences [17]. A combination of multiple temporal resolutions was used to improve the representation of physiological signals.

Hybrid architectures have also evolved to combine the advantages of CNNs and transformer-based models. VidFormer [18] integrates 3D convolutional neural networks with transformer layers to jointly model local and global dependencies. This design uses the inductive biases of convolutional layers and the attention mechanism of transformers. Another recent work, RhythmFormer [19], presents a periodic sparse attention mechanism. Their model applies a pre-attention stage to learn irrelevant periodic patterns. They improve the extraction of BVP signals in controlled environments.

In addition to supervised learning approaches, self-supervised learning has also been investigated for rPPG estimation. The RS-rPPG framework [20] used contrastive learning to learn rPPG representations without requiring ground-truth PPG signals. This approach achieved promising capabilities across different datasets.

**Table 1:** Performance Comparison of Existing rPPG Methods against Medical-Grade Ground Truth.

Category	Method	Key Methodology/Technical Contribution	MAE (bpm) ↓	Ground Truth Device
Traditional	CHROM [12]	Chrominance-based projection: Uses a linear combination of R,G,B channels to eliminate motion artifacts based on a skin reflection model.	4.06	CMS50E Pulse Oximeter
	POS [13]	Plane-Orthogonal-to-Skin: Projects color signals onto a plane orthogonal to the skin tone to maximize pulse SNR.	4.08	CMS50E Pulse Oximeter
Deep Learning	DeepPhys [14]	2D-CNN: First end-to-end model using normalized frame differences and a motion-representation branch.	6.25	CMS50E Pulse Oximeter
	TS-CAN [15]	Multi-task 2D-CNN: Employs Temporal Shift Modules and attention to capture motion efficiently without 3D convolutions.	1.7	CMS50E Pulse Oximeter
	PhysNet [6]	3D-CNN: Uses spatio-temporal convolutions to extract heart rate and BVP signals directly from video volumes.	2.95	CMS50E Pulse Oximeter
Transformer	PhysFormer [16]	ViT-based: Uses spatio-temporal self-attention to model long-range dependencies in the pulse signal.	0.52	CMS50E Pulse Oximeter
	RhythmFormer [19]	Temporal Transformer: Specifically targets the periodic rhythm of the BVP signal using constrained attention.	0.5	CMS50E Pulse Oximeter

### 2.4. Robust rPPG Estimation in Real-World Conditions

Robust rPPG estimation in unconstrained environments remains an open challenge. Real-world environments contain multiple sources of interference, including illumination variation, facial motion, and compression noise. These factors can significantly distort the extracted signals. To solve these challenges, several studies have proposed methods to model the environmental interference. Work in [21] used an environmental reference to reduce the impact of global illumination variations. It attempts to separate physiological signals from global lighting variations by using non-skin regions as environmental references. Shao et al. [22] proposed a framework that introduces a global interference to improve rPPG estimation under extreme lighting conditions. The model learns to identify and suppress illumination-induced variations using the background as a reference region.

To provide a comprehensive overview of the current landscape, Table 1 summarizes the heart rate estimation accuracy of state-of-the-art rPPG methods using the UBFC-rPPG benchmark dataset [23]. The ground-truth signals in this dataset were obtained using certified medical-grade devices, specifically a CMS50E fingertip pulse oximeter. The CMS50E is a widely accepted reference, with manufacturer specifications reporting a pulse rate accuracy of ±2 bpm for healthy subjects, while certified ECG devices achieve a rated accuracy of ±1% or ±1 bpm. These medical-grade devices serve as the teacher signals (ground truth) for training and evaluating rPPG models. They are not intended as comparative baselines but as the reference standard against which all rPPG estimations are validated. The UBFC-rPPG dataset was selected as a common reference because it provides simultaneous CMS50E oximetry for all subjects, enabling a fair comparison under a standardized protocol. The MAE values reported for each method in Table 1 are reproduced from their respective original publications.

The rPPG estimation pipeline generally consists of preprocessing, feature learning, and post-processing stages. Recent transformer-based architectures have significantly improved physiological signal extraction accuracy by modeling long-range spatio-temporal dependencies. Methods such as PhysFormer, RhythmFormer, and VidFormer demonstrated strong performance using dense self-attention mechanisms and hierarchical temporal refinement strategies. However, most existing architectures still rely primarily on implicit noise suppression within the transformer backbone and use static post-processing strategies based on predefined frequency ranges.

As summarized in Table 2, recent research trends show a gradual transition from implicit feature purification toward explicit interference modeling. Early transformer-based models depended mainly on dense attention mechanisms to suppress non-physiological artifacts indirectly. More recent approaches, such as the framework proposed by Shao et al., introduced explicit illumination modeling using background reference regions. Inspired by this direction, the proposed TH-STT framework combines spatio-temporal attention with explicit disentanglement modules, including Reaction-Driven Gating (RDG) and Dynamic Anchor Locking (DAL), to improve robustness under challenging real-world conditions.

## 3. Methodology

In real-world scenarios, the observed video is affected by multiple sources of interference, including illumination variations, subject motion, and subtle skin variations in face ROIs. The rPPG estimation can be formulated as a signal disentanglement problem, where the objective is to separate physiological components from interference sources. The proposed method aims to suppress noise components while preserving the physiological dynamics. Therefore, the proposed TH-STT framework can be viewed as learning a structured decomposition of the input signal into physiologically relevant and interference-related components, guided by both data-driven learning and physiological priors.

(1) I(t)=P(t)+M(t)+L(t)+μ(t),

where *I(t)* is the observed intensity, *P(t)* is the desired physiological component, *M(t)* represents artifacts induced by rigid and non-rigid motion (expressions and head tilt), *L(t)* represents illumination variations and environmental noise, and *μ* is the quantization noise from video compression. The objective of the TH-STT framework is to learn a mapping function that disentangles *P(t)* from the interference components {*M, L, μ*}.

### 3.1. Architecture Overview

The proposed model is a multi-task architecture that uses auxiliary task heads to improve signal reconstruction accuracy. Facial rPPG signals are corrupted with illumination and environmental interference. Interfered facial signals and environmental interference are estimated. Therefore, facial signals are weighted and separated from the environmental interference to extract robust pulse estimation.

Unlike previous transformer-based methods, the proposed framework adopts a **bimodal tokenization** strategy in which both facial regions and background regions are processed within a shared transformer backbone. This design enables feature disentanglement between physiological signals and environmental interference. Furthermore, the model proposes a **reaction-aware spatial gating** and an **adaptive frequency window** to suppress facial motion and improve signal estimation.

The proposed architecture builds upon principles of the Multi-Task Transformer [24]. The shared attention-based backbone jointly models heart rate dynamics, facial geometry, and environmental lighting to achieve accurate separation of the HR signal from noise. Furthermore, TH-STT adopts a Bimodal Patch Embedding strategy following the spatio-temporal tokenization approach introduced in ViViT (model 3) [25] and the Multimodal Token Fusion framework [26,27].

**Figure 1:**
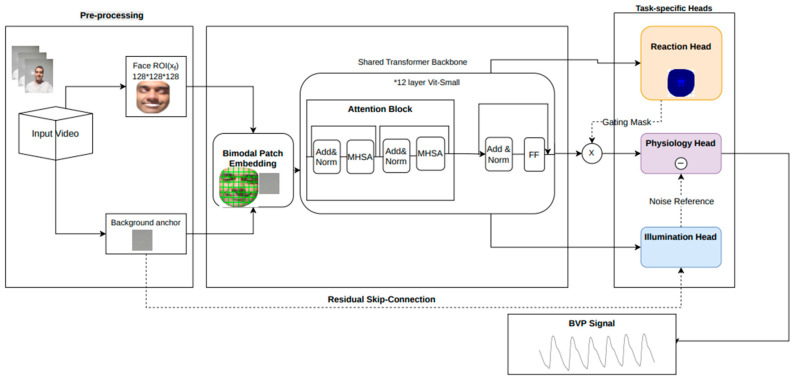
TH-STT architecture overview. Raw video frames are split into 64 facial patches and one background anchor token. A shared ViT encoder processes all tokens. Three task heads operate in parallel: Reaction Head predicts a spatial gating mask; Illumination Head extracts environmental noise via Dynamic Anchor Locking; Physiology Head subtracts the illumination signal from gated features to reconstruct the clean Blood Volume Pulse (BVP). Orthogonality loss enforces signal disentanglement.

As shown in Figure 1, the framework begins with raw video frames. Two types of tokens are extracted: a set of facial patches X_f_ and a background anchor patch X_a_. The background token serves as an environmental reference. These tokens are processed by a shared Transformer Encoder to extract global features Z. The architecture branches into three specialized task heads, each of which has an auxiliary task to achieve feature disentanglement. The Reaction Head generates a Dynamic Spatial Mask, which represents the facial muscle activity. In parallel, the Illumination Head detects environmental noise through Dynamic Anchor Locking (DAL) to provide a Noise Reference. These control signals modulate the Physiology Head, which performs gated feature suppression and signal subtraction to isolate the clean signal of blood volume pulse, S_bvp_. The system is jointly optimized via a composite loss function including an orthogonality constraint L_ortho_.

### 3.2. Dynamic Background Anchor Selection

An adaptive temporal anchor is implemented as a selection strategy during the pre-processing phase. The background anchor is used to ensure the Illumination Head receives a pure illumination reference. The anchor region is selected as a 32 × 32 pixel window from the upper region of the frame to maintain environmental representation. To align this ROI with the Transformer’s 16 × 16 patch embedding layer, we apply an area-interpolation down-sampling. This sampling strategy ensures that the selected token provides a spatially stable and robust illumination reference, avoiding the noise sensitivity of smaller windows and the inclusion of unrelated objects often encountered in larger 64 × 64 regions. The region is chosen based on stability (low spatial gradients) and minimal temporal variation. The background area is initialized using the first 128 frames of the video and is dynamically reselected every Δt = 128 frames. During each Δt interval, the system calculates the mean spatial intensity of this window as the illumination ground truth I. This adaptive update mechanism allows dynamic adaptation to changing environmental conditions.

A stability score is assigned to each candidate window in the background top area using temporal intensity variance and spatial gradient magnitude. Score is calculated as:

- Temporal Variance (*Var_t_*): measures the stability of a region over time.

(2) $$Var_{t}=\frac{1}{T}\sum_{t=1}^{T}{\left(I\left(t\right)-\bar{I}\right),}^{2}$$

where *I(t)* is the mean intensity at this window at time t, and $\bar{I}$ is the mean intensity over the patch.

- Spatial Gradient (*Grad*): ensures that the anchor is a uniform surface (like a wall) rather than a textured object. It is defined using the L2 norm of the Sobel operators *G_x_* and *G_y_*:

(3) $$Grad\left(x,y\right)=\sqrt{\left\{G_{x}^{2}+G_{y}^{2}\right\}}.$$

- Stability Index: The final score S_a_ is a weighted minimization objective:

(4) $$S_{a}={ѡ}_{1}{Var}_{t}+{ѡ}_{2}Grad,$$

where ${ѡ}_{1}$ and ${ѡ}_{2}$ are the weights of temporal and spatial variance. The region with the lowest stability score is selected as the background anchor patch. A significant challenge in rPPG-based illumination separation is the absence of ground-truth signals for environmental noise. Consequently, the stability score weights in Equation (4) were selected based on the heuristic that temporal consistency (${ѡ}_{1}$ = 0.7) is a stronger indicator of background illumination stability than spatial uniformity (${ѡ}_{2}$ = 0.3). However, depending on temporal stability may lead the model to select high-contrast textured regions that introduce edge noise during subject movement, a phenomenon observed in dynamic datasets like VIPL-HR. By involving the spatial stability weight ${ѡ}_{2}$ as a tuning factor, the system ensures the anchor is both temporally consistent and spatially homogeneous. This selection-based approach ensures that the background anchor provides a spatially stable and robust illumination reference, avoiding the noise sensitivity of smaller windows while remaining computationally efficient.

### 3.3. Spatio-Temporal Transformer Encoding

The facial and background anchor tokens are concatenated along the sequence dimension, forming a unified input. The unified input is used by the subsequent Multi-Head Self-Attention layers to perform spatial inter-stream modeling. It identifies the common illumination noise across both the physiological and environmental tokens. Let the input RGB video be represented as V ∈ ℝ^(T×H×W×C)^ where T denotes the number of frames (128), H and W represent spatial resolution, and C is the number of RGB channels (3). Face skin region is cropped at 128 × 128 pixels and partitioned into 8 × 8 patches. These patches are then projected into a D-dimensional space, forming tokens $\;X\in R^{\left(\frac{T}{T^{\prime }}\right)\times N\;\times D}$. The initial stage utilizes Bimodal Patch Embedding to partition the input into non-overlapping patches of size P × P × T′, where P = 16 and T′ denotes the temporal duration. The positional embedding is added to preserve the spatial identity of the inputs. Facial tokens are assigned numbers from 0 to 63, and the background token is assigned the 64th. Temporal positional embedding is also added to preserve the temporal signal. Placing a background token at the end of the sequence facilitates global contextual reasoning. These patches are categorized into Facial Tokens X_f_ and the specific Anchor Token X_a_, extracted from the background. Each patch is linearly projected into a D-dimensional embedding space, where D = 384, following the ViT-Small [25] standard. The architectural design was specifically engineered to overcome the limitations in current state-of-the-art rPPG transformers. Temporal-difference transformers improve motion robustness by analyzing inter-frame variations. However, this strategy may discard anatomical information required by the proposed Reaction-Driven Gating (RDG) module for accurate facial motion modeling. Similarly, although Swin Transformers offer computational efficiency through shifted window attention, their localized receptive field restricts the Dynamic Anchor Locking (DAL) mechanism. DAL requires global self-attention to correlate skin regions with distant non-physiological noise anchors. The proposed task heads require processing all frames. While using a standard ViT-Base architecture to achieve this global context is expensive for clinical or mobile deployment due to its fundamental computational requirements. Consequently, we adopted the ViT-Small backbone configuration, which provides an optimal trade-off using factorized self-attention applied to spatio-temporal embedding tubelets. This allows the TH-STT to process the real-time sequences (20–30 fps) necessary for precise physiological monitoring.

### 3.4. Reaction-Driven Spatial Gating

In the context of rPPG, facial muscle movements cause local skin distortions and shading changes. Therefore, these variations can distort the extracted physiological signal. By modeling face muscle activations, the TH-STT architecture can separate reaction-related variations from blood volume changes. It can be used to improve the accuracy of HR estimation by suppressing the effect of motion-corrupted regions.

To suppress distortions caused by facial motions, a Reaction-Driven Gating (RDG) module is proposed. It uses the concept of Spatial Gating [28] and Dynamic Masking [29,30]. The facial tokens are weighted by an importance mask, M_s_. Unlike previous static skin-masking techniques in rPPG, our mask is dynamically modulated. To model changes in facial geometry, the proposed framework generates a gating mask supervised with facial blendshape coefficients. Facial blendshapes provide the representation of facial muscle activity (e.g., Jaw-Open, Mouth-Smile).

In the training stage, the ground-truth blendshapes are obtained using the MediaPipe pipeline Face Landmarker [31]. MediaPipe employs a lightweight neural network that detects facial landmarks. It is used during the training phase to supervise the estimation of localized facial activity. Although blendshape supervision cannot perfectly model all motion-induced distortions, it provides a lightweight approximation of localized facial activity suitable for spatial gating. For each frame, a 52-coefficient vector B = [B1, B2, …, B52] is computed to represent facial muscle activations. Average activation values are then used to construct the gating mask. A pre-defined mapping matrix A∈R^52×8×8^ is used, which associates each coefficient with specific face regions in the 8 × 8 grid. Each grid cell is associated with blendshape-dependent weights according to the affected facial region. For example, if the i-th blendshape corresponds to the nose, the corresponding entries in the ith row of A in the regions in the middle are set to 1.

Using these coefficients, the model constructs a dynamic spatial mask that suppresses unstable facial regions:

(5) $$M_{s}\left(x,y\right)=1-\sum_{i=1}^{52}\bar{B_{i}}A_{i}\left(x,y\right)\;\;\;A_{i\left(x,y\right)}\in \left\{0,1\right\},$$

where $A_{i}\left(x,y\right)$ is the pre-defined spatial map value for the i-th blendshape for the region located at coordinates (x,y), and $\bar{B_{i}}$ is the average blendshape value during the batch period. A_i_ indicates whether the i-th blendshape affects the patch at (x,y).

The Reaction Head is designed as a point-wise Shared Multi-Layer Perceptron (MLP). A three-layer MLP with architecture (384-128-64-1) is used to map individual spatio-temporal tokens to mask stability scores. Our approach treats each of the 64 tokens as an independent observation with its stability score, which depends on facial activity. The reaction head is trained on the generated mask, and after the training stage, it should produce the 8 × 8 gating mask that represents facial muscle activity. To ensure robust convergence, the Reaction Head is initialized with a Unit-Bias, forcing the initial spatial mask values M_s_ = 1. This approach ensures that the physiology head initially scans the entire facial region to estimate a baseline heart rate signal. As training progresses, the reaction head reduces the effect of regions where the MediaPipe ground-truth mask detects facial movements. Calculated mask is applied to the token sequence Z to eliminate noisy tokens:

(6) $$Z_{gated}=Z⊙M_{s}.$$

**Figure 2:**
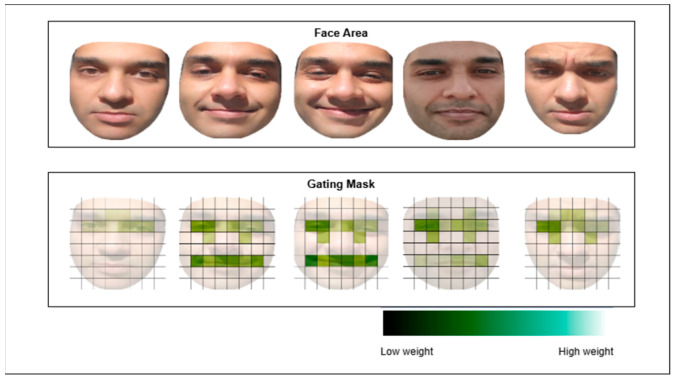
Reaction-driven spatial gating masks across different facial expressions. For each input face, an 8 × 8 spatial mask is generated based on blendshape coefficients representing facial muscle activity. Each cell corresponds to a facial patch, where lower weights suppress motion-sensitive regions such as the eyes and mouth, while higher weights preserve stable skin regions.

As shown in Figure 2, the mask values are negatively correlated with the facial activity. Accordingly, this forces the transformer to decrease the effect of regions of high anatomical variance. This gating mechanism helps the transformer to learn the representation of facial muscle activity and improves the reliability of the spatial gating mechanism described in Section 3.2. Regions strongly affected by facial reactions, such as the mouth during speech or the eyelids during blinking, can therefore be down-weighted when extracting physiological signals.

### 3.5. Illumination Modeling and Physiological Signal Reconstruction

To isolate the physiological signal, the Physiology Head H_phys_ performs the final physiological signal reconstruction. It suppresses illumination interference through feature-level subtraction, the background-derived illumination embedding. To preserve raw environmental noise, the Illumination Head used a residual Skip Connection from the input background anchor. This allows the transformer backbone to extract the temporal signal from the background anchor X_a_. Transformer attention layers may smooth high-frequency intensity changes essential for noise cancelation. Using signal skipping directly to the illumination head, raw environmental signals are included. The illumination input Z_illum_ is a fusion of attention and raw background signals:

(7) $$Z_{illum}=X_{a,layer12}+W_{skip}\left(X_{a,initial}\right),$$

where $X_{a,initial}$ is the initial background anchor embedding, and $X_{a,layer12}$ is the output of the final transformer layer. The W_skip_ is a learnable linear projection used to map $X_{a,initial}$ into the same embedding space as $X_{a,layer12}$ (nn.Linear layer). The illumination head is optimized to output the I_clean_, a 1D signal that represents the illumination variations.

To decouple environmental noise, the clean illumination signal from the Illumination Head is fed to the subtraction node. Moreover, the Physiology Head receives the gated facial tokens and applies the gated mask. The purified physiological representation is calculated by:

(8) $$Z_{phys}=W_{p}\left(Z_{gated}\right)-W_{i}\left(I_{clean}\right),$$

where W_p_ and W_i_ are learnable projections initialized with a value of one. These learnable projections are to allow the model to learn the reflectance difference between facial skin regions and the background.

To extract the heart rate signal, the frequency response H(F) is computed using a Fourier transformation:

(9) $$H(F)=\sum_{t=0}^{T}s\left(t\right)e^{-j\left(2\pi Ft+ϴ\right)},$$

where F denotes the frequency variable, ϴ is the phase offset, and s(t) represents the reconstructed physiological signal generated by the Physiology Head. As illustrated in Figure 1, the illumination signal extracted from the anchor token is subtracted from the gated features to obtain the purified physiological representation. This decoupling strategy suppresses global illumination variations.

### 3.6. Adaptive Frequency Window

Heart rate estimation in rPPG is commonly performed in the frequency domain. HR is estimated by detecting the dominant peak of the Power Spectral Density (PSD) [4] within a predefined frequency range. Most existing methods use a fixed band-pass range. To suppress non-physiological interference, the extracted BVP signal is filtered using an Adaptive Frequency Window. While the broader spectrum of cardiac electrical activity can range from 0.01 Hz to 100 Hz in traditional electrocardiography (ECG), rPPG-based heart rate estimation focuses on the fundamental frequency of heart rate. Therefore, our frequency window is constrained to the human heart rate range of 0.7 Hz to 4.0 Hz (equivalent to 42–240 bpm). This range effectively encompasses nearly all healthy and pathological heart rate variations while filtering out low-frequency DC offsets from illumination changes and high-frequency camera sensor noise. Several recent methods, including RhythmFormer [19] and Physformer [17], use a narrower filtering range (e.g., 0.75–2.5 Hz) during post-processing. Although effective under specific constrained dataset conditions, such ranges may restrict the detection of higher heart rates and limit generalization across dynamic environments. In contrast, our model tries to cover the full physiological range.

Heart rate varies depending on physical activity and emotional state. Physical activity can substantially increase the heart rate, sometimes to twice the resting level. Other activities, less strenuous, such as talking and social interaction, also activate the nervous system [32]. Therefore, the lower bound of the frequency fmin is dynamically adjusted according to facial activity intensity. Furthermore, the upper bound is constrained based on physiological limits. The maximum heart rate is commonly approximated by the relation (HRmax = 220 − age), as introduced by Fox and Haskell [33]. Accordingly, the upper frequency bound is fixed at 210 bpm (3.5 Hz) to suppress non-physiological high-frequency noise.

An adaptive frequency window is proposed that dynamically adjusts the lower bound of the spectral search range based on facial activity, while maintaining a fixed upper bound:

(10) $$fmin\left(t\right)=0.7+c\cdot \psi \left(t\right),\;fmax=3.5,$$

where the Global Activity Index (ψ) is used as the mean change in intensity of the active gating mask. The parameter c controls the degree of the lower frequency bound. We further analyze the sensitivity of the hyper-parameter c in the Adaptive Frequency Weighting (AFW) module. Physiologically, intense facial activity is often associated with elevated sympathetic nervous system activation and heart-rate variability [34]. From an engineering perspective, high facial activity (e.g., speaking or emoting) introduces low-frequency motion artifacts into rPPG signals. Increasing fmin during periods of high facial activity helps suppress motion-induced interference. Sensitivity analysis shows that the model is robust within a reasonable range of c values (e.g., 0.1–0.4), with only minor performance differences. Results of different values are added to the ablation study. The hyper-parameter c = 0.2 was determined through a sensitivity analysis across the VIPL-HR dataset to balance motion-artifact suppression while preserving low resting heart rates. The final HR is calculated by locating the frequency peak within the adaptive window of the Power Spectral Density (PSD):

(11) $$HR=60\cdot {argmax}_{f\in \left[fmin,fmax\right]}.PSD(s(t)),$$

where PSD denotes the Power Spectral Density of the predicted physiological signal at frequency f, and argmax [35] identifies the frequency with the maximum spectral power within the adaptive range [fmin, fmax]. A scaling factor of 60 is applied to convert the estimated frequency from Hz to beats per minute (bpm).

### 3.7. Multi-Head Objective Functions and Optimization

The model is optimized using a joint multi-task loss [26]. Auxiliary tasks are incorporated during training to improve the generalization of the primary physiological reconstruction task by providing a more inductive bias [36]. The multi-task loss function helps in the simultaneous optimization of physiological reconstruction, facial activity estimation, and illumination modeling.

In this architecture, each head is supervised by a specialized loss function to learn a specific task. This approach guarantees that the shared backbone develops attention focus on pulse, facial muscle movements, and lighting data. The total loss function L_total_ is calculated as a weighted sum of specialized loss functions:

(12) $$L_{total}=\lambda_{phys}\cdot L_{phys}+\lambda_{react}\cdot L_{react}+\lambda_{illum}\cdot L_{illum}+\lambda_{ortho}\cdot L_{ortho}$$

The weighting coefficient values are dynamically adjusted in the training stage using progressive scheduling. Progressive scheduling and loss coefficient values are discussed in Section 4.2 and Section 4.4. Coefficients are experimentally tuned to balance the gradient magnitudes and prevent the specialized tasks from dominating the main task [22].

#### 3.7.1. Physiology Loss (L_phys_): Hybrid Time-Frequency Supervision

Although Mean Squared Error (MSE) effectively supervises signal reconstruction, it is sensitive to amplitude variations between the predicted and ground-truth signal. In rPPG applications, amplitude variations can occur due to differences in skin tone and illumination conditions. To address this limitation, a negative Pearson correlation loss [6] is used to emphasize similarity in waveform shape rather than absolute amplitude. PhysNet [6] and PhysFormer [16] demonstrated that correlation-based and negative Pearson correlation loss improve waveform reconstruction accuracy.

The Physiology Head is optimized to reconstruct the BVP signal s(t). Following the standards set in PhysFormer++ [17], a hybrid loss is used to enforce both morphological accuracy in the time domain and periodic consistency in the frequency domain:

(13) $$L_{phys}=(1-\rho (S,g))+\alpha \cdot CE(PSD\left(S\right),f_{gt}),$$

where f_gt_ denotes the ground-truth heart rate frequency, $\rho \left(S,g\right)$ represents the Negative Pearson Correlation between the predicted signal S and ground truth g. To enforce consistency between the predicted spectrum and the ground-truth heart-rate f_gt_, Spectral Cross-Entropy (CE) [37] is applied to the Power Spectral Density (PSD) [16]. The spectral distribution is normalized and treated as a probability distribution before applying cross-entropy loss. Hyper-parameter values were set as α = 0.5 to balance spectral with temporal accuracy. It prevents the model from falling into local minima of predicting the correct frequency with an incorrect pulse shape.

#### 3.7.2. Reaction Loss (L_react_): Anatomical Distillation

The Reaction Head is trained using the 8 × 8 gating mask generated from MediaPipe facial activity estimation [31]. The module predicts a token-wise stability score for each of the 64 tokens. The loss is defined as the average Binary Cross-Entropy (BCE) across the spatial manifold:

(14) $$L_{react}=\frac{1}{N}.\Sigma -[M_{gt,i}.\log\left(M_{i}\right)+(1-M_{gt,i}).(\log\left({1-M}_{i}\right))],$$

where M_gt,i_ is the ground truth of the ith token, and Mi is the predicted gated value. By minimizing this difference, the model learns to identify motion-sensitive facial regions affected by muscular movements.

#### 3.7.3. Illumination Loss (L_illum_): Background Reference

The Illumination Head captures the illumination artifact from the selected background anchor. Using the residual skip connection, the Illumination Head is trained using a self-supervised consistency constraint. The average temporal intensity of the background anchor ROI is used as a physical reference for illumination noise. The head is optimized to generate an illumination signal I_clean_ that maximizes the Pearson Correlation with the background mean intensity signal:

(15) $$L_{illum}=1-\frac{conv(I_{clean},Y_{anchor})}{\sigma_{\mathrm{I\_}}.\sigma_{Y}},$$

where I_clean_ is the output of the illumination head, and the Y_anchor_ is a 1D signal representing the average temporal intensity of each of x_a_ frames.

#### 3.7.4. Orthogonality Loss (L_ortho_): Latent Disentanglement

Orthogonality loss is used for separation between the physiological signal and environmental noise. Orthogonality Loss based on the self-supervised disentanglement proposed by Shao et al. [22]. The orthogonality constraint minimizes the squared correlation between the reconstructed pulse s(t) and the illumination vector I_clean_(t):

(16) $$L_{ortho}=(Cov(S,I_{clean})/(\sigma_{S}.\sigma_{I}){)}^{2},$$

where Cov (S, I) represents the covariance between the signals, while σ_S_ and σ_I_ denote their standard deviations. Orthogonal loss acts as a controller for back-propagation. It forces the Physiology Head and Illumination Head to adjust their features independently.

### 3.8. Lightweight Auxiliary Heads

Although the proposed architecture includes multiple task heads, the additional heads are designed to be lightweight task components. Their primary role is to provide supervision for learning. These heads do not significantly increase the computational complexity of the model.

All auxiliary task heads operate on global pooled transformer features. Therefore, their computational complexity is negligible compared to the transformer backbone. The dominant computational cost of the architecture originates from the self-attention operations within the transformer encoder, at which Complexity scales with the number of tokens and attention heads. In contrast, the task heads introduce only a small number of additional parameters corresponding to the fully connected layers.

During training, the task heads provide supervision training signals. Their associated losses guide the model toward accurate estimations. During execution, these heads generate lightweight predictions with minimal impact on runtime performance.

To distinguish the proposed TH-STT framework from existing multi-component and transformer-based strategies in the rPPG literature, Table 3 compares the design philosophy of recent rPPG approaches. Traditional CNN-based models, such as MTTS-CAN and PhysNet, utilize multi-task or multi-stream architectures for passive feature fusion and motion regularization. However, they do not have the ability to dynamically re-prioritize spatial features during inference. Similarly, recent Transformer benchmarks like VidFormer and the framework by Shao et al. use sophisticated attention mechanisms for feature extraction. However, VidFormer remains primarily noise-agnostic, relying on dense global attention without explicit disentanglement. Additionally, Shao et al. depend on visual similarity between the subject and the background. Our proposed framework introduces more interaction between auxiliary supervision and physiological feature extraction. The proposed TH-STT framework differs from prior multi-component architectures by integrating facial dynamics directly into the physiological feature extraction process.

Unlike prior multi-component architectures that treat task heads as independent outputs, the TH-STT utilizes the Reaction-Driven Head as a real-time control mechanism for the Physiological Head. Specifically, the Reaction Head computes a spatial importance map, which acts as a dynamic weighting schema. The generated spatial importance map allows the model to reduce emphasis on motion-sensitive regions (e.g., the mouth during speech) to stable anatomical ROIs. The TH-STT offers spatial flexibility and noise robustness that is difficult to achieve with the static, uniform attention mechanisms of models such as VidFormer or PhysFormer by associating the noise-awareness of the Reaction and Illumination Heads directly to the feature-extraction process.

## 4. Experimental Results

### 4.1. Datasets

To evaluate the accuracy of our TH-STT implementation, three benchmark datasets that represent the most real-world challenges, from extreme motion to volatile illumination, were used. Dataset details are shown in Table 4. The datasets were collected following ethical guidelines, with informed consent from all participants and approval from the relevant ethics committee.

- UBFC-rPPG [23]: Designed to induce high heart rate variability (HRV) and natural facial expressions, including 42 participants playing a stress-inducing math game. Videos are recorded using a Logitech C920 HD Pro webcam at 640 × 480 resolution at 30 fps. The capturing environment is indoor with good illumination, with a varying amount of sunlight. All videos have a negligible compression (220 Mbps) to preserve subtle physiological variations.
- VIPL-HR [38]: A multi-modal dataset containing 2378 videos from 107 subjects. It includes 9 different scenarios, including varied head movements and three different video sensors (webcam, smartphone, and NIR). RGB videos are captured using a Logitech C310 color camera (960 × 720 at 25 fps) and a Huawei P9 camera (1920 × 1080 at 30 fps). The environments include dark and bright illumination with moderate compression. VIPL-HR contains substantial motion variation, sensor diversity, and illumination inconsistency, making physiological reconstruction significantly more challenging than UBFC-rPPG.
- COHFACE [39]: It contains 40 subjects with different age groups and skin tones. Each subject is recorded in four video sequences, under two experimental conditions: clean (controlled lab illumination) and natural (lights off, only sunlight entering the lab). Videos are recorded using Logitech HD C525 640 × 480 resolution at 20 fps. All videos are stored with a high compression rate (250 kbps), which leads to compression artifacts that can hide the subtle skin color variations and increase the difficulty of accurate rPPG estimation.

### 4.2. Implementation Details

The proposed TH-STT framework is implemented using PyTorch 2.12 and executed on a NVIDIA RTX 4090 GPU. The shared spatio-temporal backbone consists of 12 transformer layers (L = 12). To balance between computational efficiency and model capacity, dimensionality of D = 384 is used, inspired by the ViT-Small configuration [25]. ViT-Small uses a reduction from the standard D = 768 (ViT-Base) to ensure real-time performance on all devices and to prevent over-fitting. All experiments follow that training and testing subjects are disjoint. The network is optimized using the AdamW optimizer with an initial learning rate of 1 × 10^−4^ and decreased to 10^−5^ after 50 epochs for the last epoch.

Data partitioning for smaller datasets, such as UBFC-rPPG and COHFACE, a 5-fold subject-independent cross-validation protocol was used. This ensures that every subject in the smaller cohorts is used for evaluation. Conversely, the large-scale VIPL-HR dataset follows an 80/20 subject-independent protocol to ensure unbiased evaluation and the generalization to physiological signatures. A subject-independent random split (80% training, 20% testing) was used. Specifically, subjects were randomly assigned to the training and testing sets by their IDs. Individual subjects that present during the optimization phase do not appear in the evaluation phase. To account for training variance, the results are reported as the mean of three independent trials with different random seeds.

To ensure stable convergence and mitigate gradient imbalance in the proposed multi-task framework, we adopt a three-stage curriculum learning strategy combined with a tiered learning rate schedule. This design allows the shared transformer backbone to progressively learn robust spatial and temporal representations before addressing the more challenging task of illumination disentanglement.

The curriculum is motivated by two key observations. First, the illumination loss tends to dominate the optimization process during early training due to the strong correlation between background intensity signals and global luminance variations, which can suppress the learning of subtle physiological features. Second, the reaction loss is comparatively easier to optimize, as it relies on structured blendshape supervision and therefore provides a reliable inductive bias for initializing spatial attention. Accordingly, the training proceeds in three stages:

- Stage 1 (Epochs 1–10: Spatial Prior Initialization).

The model is trained using the Reaction and Physiology losses, with λ_react_ = 1.0 and a reduced weight for λ_phys_, while λ_illum_ and λ_ortho_ are disabled. This stage encourages the model to first learn stable spatial attention patterns and region reliability, preventing premature overfitting of the physiological signal to noisy representations. The learning rate is set to 1 × 10^−4^.

- Stage 2 (Epochs 11–25: Physiological Signal Learning).

The learning process is shifted to the Physiology loss (λ_phys_ = 1.0) while keeping a reduced weight from the Reaction loss. This residual weighting serves as a regularization mechanism, preserving previously learned spatial information and preventing catastrophic forgetting. The learning rate is kept at 1 × 10^−4^ for efficient adaptation.

- Stage 3 (Epochs 26–100: Full Multi-task Refinement).

All loss terms are activated. The loss of illumination is added with a relatively small weight coefficient to avoid it dominating the optimization. Since illumination signals exhibit higher magnitude and strong correlation with pixel intensity, they can otherwise overwhelm the physiological signal. To further stabilize training, the learning rate is reduced to 5 × 10^−5^ for the first 15 epochs of this stage, and subsequently to 1 × 10^−5^ for the remaining epochs. This progressive annealing makes the orthogonality constraint effective in enforcing disentanglement while ensuring stable convergence and alleviating overfitting. This curriculum corresponds to gradually increasing the task complexity, from the reaction-aware spatial modeling (well-supervised and stable) to the physiological signal reconstruction (requiring temporal consistency) and finally to the illumination disentanglement (most sensitive as it can suppress the target signal). Hyper-parameters, including stage durations, learning rates, and loss weights, were selected via staged grid search as described in Section 4.4.7.

### 4.3. Results and Discussion

Performance of our proposed TH-STT is evaluated against several models, including Traditional methods such as CHROM and POS, Deep-learning models like TS-CAN, DeepPhys and PhysNet and transformer models like PhysFormer, PhysFormer++, RhythmFormer, and RS-rPPG. Table 5 shows the Mean Absolute Error (MAE) and Root Mean Square Error (RMSE) across three benchmark datasets. Best results are bold and underlined.

On datasets containing emotional and human interaction, such as UBFC-rPPG, TH-STT achieves better accuracy (MAE of 0.42). The Reaction Head and gating mechanism filter out regions associated with emotional micro-expressions. Our model, using the generated spatial mask, suppresses motion-corrupted facial regions during physiological signal extraction. Furthermore, the Adaptive Frequency Window (AFW) dynamically adjusts its pass-band using the activity signal produced by the Reaction Head.

Our model’s performance on the COHFACE dataset is remarkable, especially given its high compression rate. While video compression typically introduces quantization distortions that increase the blurring of the finest skin tone details, our model achieved an average absolute error (MAE) of 1.08 beats per minute (bpm). The proposed model handles most low-light situations and facial movements with high accuracy. In some cases, with numerous sources of noise, the error increases, and this is discussed in the failure case study section. Unlike UBFC-rPPG, where illumination is stable, COHFACE’s natural condition relies solely on sunlight, which our DAL mechanism effectively compensates. The strong performance under compression-heavy conditions suggests that the proposed disentanglement strategy remains stable even when fine-grained skin-tone variations are partially degraded.

TH-STT maintained robust performance on the VIPL-HR dataset despite large motion and environmental variability. Our model achieves a competitive MAE of 4.65 bpm, which remains comparable to recent state-of-the-art transformer-based approaches. The VIPL-HR dataset presents extreme challenges: rapid, large-amplitude head movements, diverse sensors (webcam, smartphone, NIR), and illumination ranging from very dark to bright. Under such conditions, our TH-STT achieves a MAE of 4.65 bpm—slightly higher than RhythmFormer’s 4.51 bpm—but a significantly better RMSE of 7.23 bpm versus 7.98 bpm. This discrepancy reflects our model’s strength: the explicit illumination head (DAL) effectively suppresses lighting variations across dark and extreme illumination scenarios, reducing large errors (outliers) that inflate RMSE. However, the fast, large motions remain challenging for any method; the slightly higher MAE indicates that motion artifacts are not yet fully eliminated, which we discuss in the failure case analysis.

To better understand the observed performance differences, we further compare the behavior of transformer-based and non-transformer architectures. Compared to non-transformer state-of-the-art (SOTA) baselines, the results in Table 5 show a consistent pattern: transformer-based methods (PhysFormer, RhythmFormer, TH-STT) achieve substantially lower MAE than traditional (CHROM, POS) and CNN-based (DeepPhys, PhysNet, Ts-CAN) approaches, particularly on challenging datasets (VIPL-HR, COHFACE). This performance gap directly reflects the theoretical limitations discussed in Section 2.1 and Section 2.2. Traditional methods remain limited because their fixed signal transformations cannot adapt to variable lighting or motion. As discussed before, they lack the capacity to model the complex relationship between compression noise and the physiological signal. Methods like CHROM and POS exhibit MAE values exceeding 6 bpm on COHFACE. CNNs suffer from limited temporal receptive fields (unable to capture multiple cardiac cycles) and lack explicit noise disentanglement. Even CNN-based architectures like PhysNet were optimal with the long-range temporal dependencies required to distinguish heart rate from quantization noise. However, when test conditions shift (e.g., VIPL-HR’s cross-sensor variability), implicitly learned noise suppression collapses. Transformers models addressed these limitations through self-attention over long sequences (100+ frames), dynamic spatial prioritization (e.g., attending to forehead over mouth), and the ability to reference background tokens for explicit illumination estimation—as demonstrated by our DAL mechanism.

Compared to self-supervised Rs-rPPG, using the VIPL-HR dataset, RS-rPPG’s performance is lower than that of supervised methods. VIPL-HR poses many challenges, which complicate the reconstruction of physiological signals without explicit supervision. Although self-supervised methods reduce the reliance on ground-truth annotations, they may be limited in distinguishing complex environmental interference in highly dynamic scenarios. Self-supervised models cannot handle dynamic noise effectively. In contrast, the supervised models provide implicit/explicit guidance for motion suppression and illumination disentanglement, thus improving signal stability under challenging recording conditions.

As discussed in Section 3.6, PhysFormer and RhythmFormer used a narrower band-pass filter based on dataset-specific HR distributions. PhysFormer used a range between 42 and 180 bpm and removed samples with higher values from the VIPL-HR dataset. Similarly, RhythmFormer stated that they used a band-pass filter with a range from 0.75 to 2.5 Hz, which corresponds to 45–150 bpm. Using a narrower frequency range and excluding a part of the dataset may improve model performance and eliminate the noise effect. However, this will not be the case in a dynamic environment.

The observed performance improvements are associated with the Reaction-Driven Dynamic Spatial Gating. Traditional transformers may include signals resulting from unstable patches merging with the physiological signal. Our Reaction Head identifies face reactions as a gating signal, allowing the model to suppress noisy tokens. Gating prevents the backbone from detecting motion-related frequency as physiological pulses.

While methods like Shao et al. used a global background reference, our model performs a subtraction of the illumination signal derived from the background anchor. Using the Orthogonality Loss (L_ortho_), our model forces the illumination and physiological signals to be independent. The network learns how to isolate the resulting BVP from flicker and low-light sensor noise.

Finally, the experimental results demonstrate that the proposed TH-STT improves the accuracy under challenging conditions. The combination of reaction-aware gating, illumination decoupling, and adaptive frequency filtering enables the model to maintain stable performance across different datasets.

### 4.4. Ablation Study

An ablation study is conducted to evaluate the individual contributions of each proposed unit within the TH-STT architecture. Our objective is to isolate the performance improvement achieved by the Reaction-Driven Gating and Illumination mechanisms under challenging conditions. We define four primary configurations for our evaluation:

#### 4.4.1. Baseline Model (M_base_)

The baseline consists of the shared transformer backbone and the Physiology Head (H_phys_) only. In this configuration, the model receives only the facial ROIs (X_f_) and performs standard pulse estimation without any external gating or noise reference, which represents a standard transformer-based rPPG baseline without explicit motion or illumination disentanglement.

#### 4.4.2. Impact of Reaction-Driven Gating (M_react_)

Reaction Head (H_react_) and the Dynamic Spatial Gating module are integrated with the backbone. This configuration tests the model’s ability to remove noisy face tokens. We evaluate this on the datasets to observe how well the model ignores noisy tokens from facial reactions like speaking or smiling.

The proposed TH-STT architecture effectively mitigates error propagation from the external blendshape extractor through a combination of high-precision priors and a phased Multi-Task Learning (MTL) strategy. First, state-of-the-art landmark detectors (e.g., MediaPipe) provide sub-pixel accuracy in localized muscle intensity extraction. MediaPipe blendshqapes itself has several limitations:

- Detection Accuracy and Temporal Jitter: MediaPipe provides sub-pixel landmark localization. However, extreme head rotations (>45 degrees) or severe occlusions (hands covering face) cause detection failures. Moreover, raw blendshape coefficients exhibit frame-to-frame jitter due to video compression and landmark tracking noise. To reduce temporal jitter, a moving average filter is applied (through the window) before computing. Additionally, the soft-gating formulation (Equation (5)) produces gradual mask changes rather than binary switching, making the system robust to small coefficient changes.
- Anatomical Mapping: The mapping matrix A (52 blendshapes to an 8 × 8 spatial grid) requires manual definition of which facial regions each blendshape affects. This is an approximation; in reality, muscle activations have diffuse effects. We minimize this limitation by: (a) using a coarse 8 × 8 grid (64 patches total), so spatial precision requirements are modest, and (b) allowing the transformer to learn residual adjustments through end-to-end training, as the gating mask is a soft weight, not a hard segmentation.

Our phased curriculum learning (Section 4.2) initializes the Reaction Head first, allowing stable spatial attention learning before full physiological reconstruction optimization. Ablation results (Table 6) show that RDG consistently improves accuracy, confirming that benefits outweigh any error propagation.

#### 4.4.3. Impact of Illumination Decoupling (M_illum_)

This configuration adds the Illumination Head (H_illum_) and the dynamic anchor locking to the backbone. Here, the model’s capacity to identify and subtract global light noise is tested. Sensitivity analysis showed minimal variation in anchor selection, with similar stable regions consistently identified across different parameter settings. To validate the selection of the start-up weights used in the stability index (w_1_, w_2_), a sensitivity analysis was performed by varying w_1_ from 0.6 to 0.9 and w_2_ from 0.1 to 0.4. The final results remained invariant; it was selecting the same window or a similar window with the same setup.

#### 4.4.4. Full TH-STT Synergy (M_full_ Without/with AFW)

The final configuration represents our complete proposed architecture. All three heads are added. Full setup evaluates the impact of reaction masking and lighting subtraction techniques. We assume that the combined optimization of these three tasks allows the shared structure to learn a more robust disentangled physiological representation. These results demonstrate that the combination of RDG and DAL improves robustness and estimation accuracy. For results without AFW, post-processing is achieved at the full band frequencies [0.7–3.0] Hz. For results with AFW, the AFW was used to adapt the band-pass filter. As shown in the results, the AFW improves the estimation accuracy by reducing the MAE by 4%.

All ablation variants are trained using the same hyper-parameters (learning rate: 10^−4^ –10^−5^, AdamW optimizer) to ensure a fair comparison. Mean Absolute Error is used as our primary metric. As shown in Table 6, by comparing these four stages, experimental evidence is provided that the interaction between task-specific heads is essential for reliable rPPG estimation in interactive environments.

On the relatively stable UBFC-rPPG dataset, the Reaction Head has a measurable gain in accuracy. This result confirms the effectiveness of the Illumination Head in handling illumination variability. The full TH-STT synergy achieves our optimal result of 0.42 bpm. This confirms that while the architecture provides a strong foundation, the auxiliary tasks are the primary drivers for isolating the BVP signal from environmental noise and facial motion.

#### 4.4.5. Impact of Temporal Window Size

During the pre-processing stage, face detection is performed using the MediaPipe framework. The identified facial Region of Interest (ROI) is cropped and resized to a fixed resolution of 128 × 128 pixels using bi-cubic interpolation. Data is processed using a sliding window of 128 frames (approximately 4.27 s at a sampling rate of 30 fps). During the pre-processing stage, the background anchor is selected for its spatial stability. It is identified in the first Δt and subsequently re-initialized every Δt to dynamically deal with non-stationary illumination.

To investigate the impact of the temporal sequence length on the model’s performance, we conducted an ablation study by varying the window size T = {64, 96, 128, 160}. Temporal window T size was selected based on the tradeoff between estimation accuracy and computational complexity. As shown in Table 7, using a window size of T = 64 is often insufficient to cover two complete cardiac cycles, which are required for stable frequency-domain analysis, especially in individuals with low heart rates (e.g., 45–55 bpm), resulting in a high root mean square error (RMSE). While setting the window size to 160 significantly improves accuracy, it essentially increases computational cost. Therefore, using a window size of 128 achieves temporal coverage for the target frequency range while maintaining a balance between accuracy and computational efficiency.

#### 4.4.6. Adaptive Frequency Window Parameter

**Figure 3:**
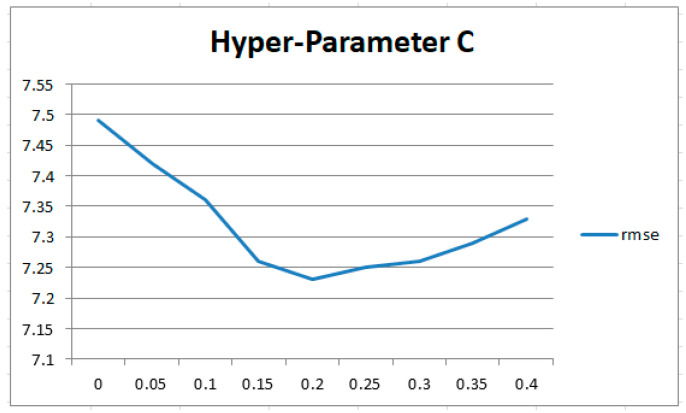
Sensitivity analysis of the Adaptive Frequency Window parameter c on the VIPL-HR dataset.

To evaluate the sensitivity of the Adaptive Frequency Window (AFW) to its primary scaling parameter, we conducted an ablation study by varying the hyper-parameter c in Equation (10) across the range [0, 0.4]. The relationship between c and the RMSE using the VIPL-HR dataset is shown in Figure 3. The model achieves its peak performance at c = 0.2, yielding the minimum RMSE of 7.23. Values below 0.2 result in higher error due to insufficient suppression of low-frequency motion artifacts resulting from high facial reactivity. Additionally, values exceeding 0.25 begin to degrade performance due to the over-shifting of the frequency floor, which may suppress valid physiological frequency components.

#### 4.4.7. Sensitivity Analysis and Parameter Selection

**Figure 4:**
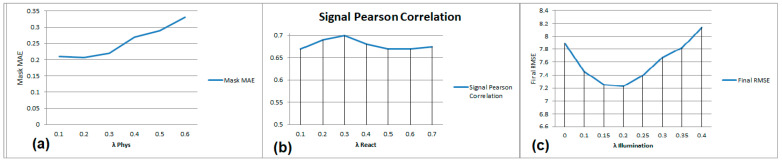
Staged grid search for multi-task loss coefficients. (**a**) Stage 1: λ_phys_ selection minimizing mask MAE. (**b**) Stage 2: λ_react_ selection balancing Pearson correlation and mask MAE. (**c**) Stage 3: λ_illum_ selection minimizing heart rate RMSE.

The TH-STT model was optimized using a three-stage curriculum learning procedure, with hyper-parameters selected via a sequential grid optimization search as illustrated in Figure 4.

- Stage 1: The initial objective was to establish a stable spatial gating mask in the first 10 epochs. We fixed the reaction coefficient λ_react_ = 1.0 and performed a grid search for the physiological coefficient λ_phys_ between 0.1 and 0.6. As shown in Figure 4a, the Mask MAE reaches its minimum at λ_phys_ = 0.2. Beyond this threshold, physiological gradients begin to dominate the latent space, leading to a degradation of the gating mask accuracy.
- Stage 2: In the second stage, we prioritized signal extraction by fixing λ_phys_ = 1.0 and searching for the optimal λ_react_ value. The selection was based on identifying the stability point between signal purity and gating stability. As depicted in Figure 4b, the highest Pearson correlation was achieved at λ_react_ = 0.3, ensuring the model preserves its learned spatial priors while maximizing BVP signal quality.
- Stage 3: The final stage introduced the illumination and orthogonality coefficients. Our analysis in Figure 4c demonstrates that the RMSE reaches an optimal value at a coefficient value of 0.2. Setting the illumination weight higher than 0.2 leads to a gradient dominance of the illumination head. At higher values for λ_illum_, the illumination head overfits illumination reconstruction by modeling light variations, effectively treating the subtle BVP signal as noise and causing a sharp rise in final error.

### 4.5. Computational Complexity

We evaluated the computational efficiency of the TH-STT against CNNs and state-of-the-art transformers using a unified hardware platform equipped with an NVIDIA RTX 4090 GPU. Computational efficiency was evaluated by measuring the average inference time per frame across different architectures. The results are shown in Table 8, which indicates that TH-STT achieves a favorable trade-off between computational complexity and execution speed.

The proposed model has the highest parameter count (14.4 M). However, its computational workload per frame (470 M FLOPs) is lower than CNN architectures like DeepPhys (744 M FLOPs). These observations suggest that sparse tokenization reduces the pixel-level redundancy associated with dense convolutional processing. Compared to other work, our model achieves the lowest RMSE (7.23) in the entire comparison group. Although PhysNet exhibits the lowest latency (<0.13 ms), it suffers from a nearly 2.x increase in RMSE compared to our model.

### 4.6. Error Distribution and Agreement Analysis

**Figure 5:**
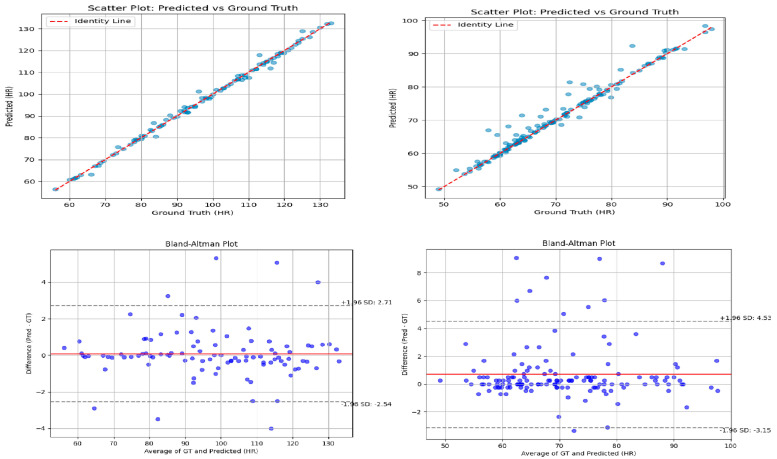
Correlation (**top row**) and Bland–Altman (**bottom row**) agreement analysis for UBFC-rPPG (**left**) and COHFACE (**right**). Scatter plots show predicted vs. ground truth heart rate with a regression line. Bland–Altman plots show bias (red line) and 95% limits of agreement (dashed lines).

To evaluate the clinical agreement and error distribution of the TH-STT, we present scatter plots and Bland–Altman analyses for the evaluated datasets (Figure 5). The scatter plots show a strong linear correlation between the ground truth (BVP) and the predicted rPPG signals (50–140 bpm).

The Bland–Altman plots quantify this agreement, showing a negligible mean bias. For the UBFC-rPPG dataset (Figure 5, left), the 95% Limits of Agreement (LoA) are remarkably narrow, ranging from −2.54 to +2.71 bpm. In a more challenging scenario, the COHFACE dataset (Figure 5, right), the LoA remains within a robust [−3.15, +4.53] bpm interval. These plots confirm that the error distribution is tightly bound, with the vast majority of estimations falling within the clinically acceptable margin. The small number of positive outliers observed is discussed in the following sub-section for the failure case analysis. This distribution confirms that the TH-STT is free from systematic bias and that the vast majority of errors fall within a clinically acceptable margin for non-contact monitoring.

### 4.7. Failure and Challenging Case Analysis

**Figure 6:**
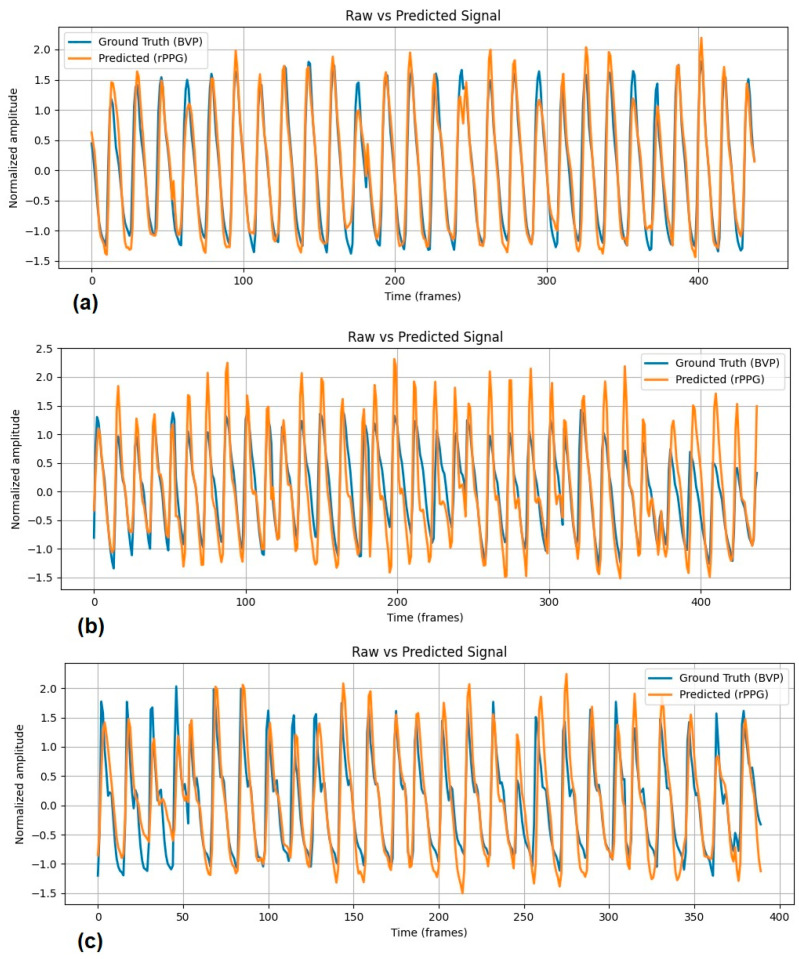
Successful rPPG estimation in challenging cases (Ground truth BVP (blue) and TH-STT prediction (orange)) over time: (**a**) Dark skin under low illumination. (**b**) High heart rate. (**c**) Elderly subject.

The TH-STT results show robustness by maintaining high signal fidelity under diverse conditions that typically degrade rPPG performance. Figure 6 presents challenging scenarios in which the proposed framework successfully tracks the BVP signal. The ground truth signal is shown in blue, and the predicted signal is shown in orange.

Dark Skin with Low Illumination (Figure 6a): The model captures the pulse rhythm for subjects with dark skin tones and in low-light environments, maintaining high peak-to-peak correspondence.

High Heart Rate Ranges (Figure 6b): The architecture maintains stable signal tracking under high heart-rate conditions. It successfully captures peaks without the damping effect of standard transformers.

**Figure 7:**
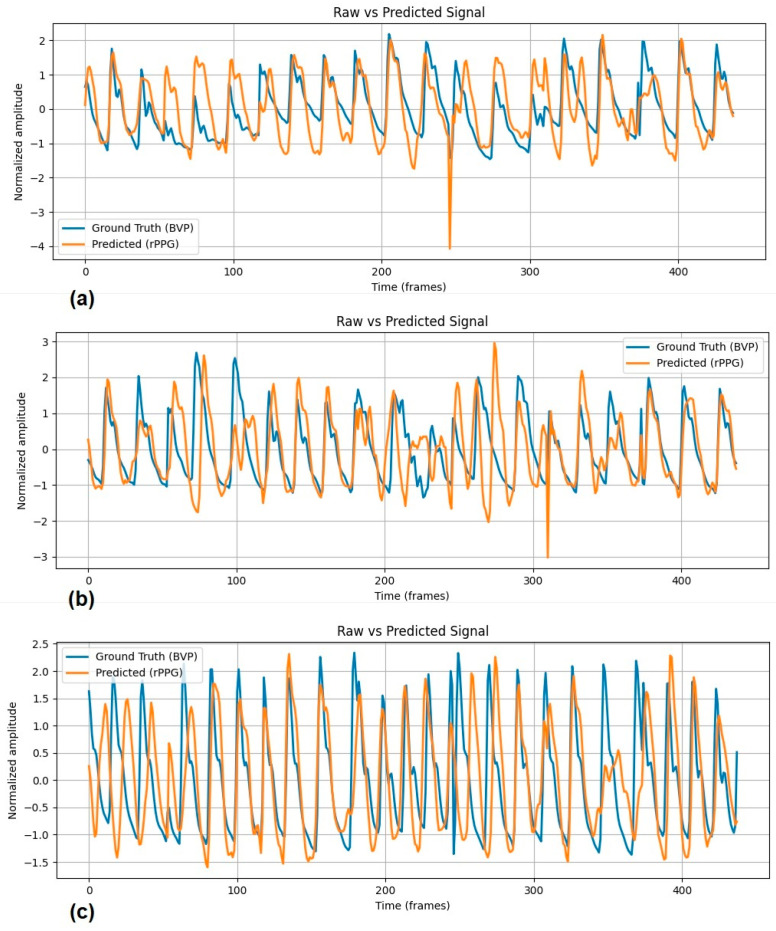
Representative failure cases. (**a**) Sudden head rotation under low illumination causes a transient artifact (**b**) Continuous head movement leading to phase drift. (**c**) Camera motion with high heart rate variability causes amplitude damping.

Elderly Subjects (Figure 7c): Despite the physiological changes in skin elasticity associated with age, the predicted rPPG signal closely follows the BVP ground truth.

While the TH-STT demonstrates high accuracy across benchmark datasets, we identify specific scenarios where the interplay of environmental noise and signal compression challenges the architecture. As illustrated in Figure 7, these sub-failures provide some edge cases. For all cases, they suffer from a high compression rate. The high compression rate of this dataset introduces quantization artifacts that can hide the subtle PPG signal.

(a) Transient Rapid Movement in Low Illumination: In Figure 7a, a sharp transient spike is observed near frame 250. This corresponds to a sudden, high-magnitude head movement under low-light conditions. Sudden movements in low illumination are hard to deal with. It is difficult to deal with due to the sudden changes in the targeted skin and the change in light reflection.
(b) Continuous Movement and Phase Drift: Figure 7b demonstrates the effect of sustained motion in low illumination. At the same time, the predicted signal maintains the correct periodicity. However, it exhibits Phase Drift misalignment with some of the ground truth peaks. This suggests that continuous motion in compressed video streams can cause the Factorized Attention mechanism to struggle with maintaining exact peak correspondence.
(c) HRV Tracking under Compounded Noise: In Figure 7c, the model faces a combination of camera motion, low illumination, and High Heart Rate Variability (HRV). The ground truth shows rapid changes in the inter-beat interval. The TH-STT successfully tracks the general trend. However, the compounded noise results in a damping effect where some specific peak magnitudes are slightly underestimated.

### 4.8. Generalization Analysis

TH-STT is evaluated on three benchmark datasets covering motion, illumination, and cross-device variability. In this setup, the model is trained on the source dataset and evaluated on another dataset without any fine-tuning. The results prove the model’s ability to handle domain shifts in skin tone, camera sensor noise, and environmental conditions.

We trained our model on the VIPL-HR dataset and evaluated it on UBFC-rPPG. The results show that our model maintains high accuracy even with shifts in environments. The results are shown in Table 9.

Improvement can be attributed to the disentanglement of physiological and non-physiological features:

- Using Dynamic Anchor Locking (DAL) technology, the model learns to isolate lighting noise and helps the lighting head to adapt to illuminating a new dataset instantly.
- The Reaction Head uses a uniform blending pattern. Since human facial muscle movements are anatomically universal, a dynamic spatial gate technique learned on one group of people can be applied to a completely different population in another dataset.
- The Orthogonality Loss (L_ortho_) ensures that the latent space is un-noisy. When moving to a new dataset, the model is not confused by new types of background noise because it has already learned that the BVP signal should be environment-independent.

### 4.9. Detailed Noise Robustness Comparison

To clarify how TH-STT achieves robust noise resistance, we analyze the specific limitation modes of existing transformer architectures. In PhysFormer, the temporal difference (TD) mechanism computes frame-to-frame residuals to emphasize motion. However, this amplifies high-frequency sensor noise and fails to distinguish between physiological pulsations and facial expression-induced color changes. The dense self-attention treats all spatial locations uniformly, so a smiling mouth corrupts the pulse signal from the forehead through global attention mixing. Moreover, using RhythmFormer, the periodic sparse attention constrains the model to learn repeating patterns, which is effective for stationary subjects. However, when illumination varies non-periodically, the sparsity mechanism discards these aperiodic changes as irrelevant, losing the reference needed to remove them. The dataset-specific band-pass filter (0.75–2.5 Hz) also excludes high heart rates (>150 bpm). Using VidFormer, the 3DCNN + transformer hybrid captures local motion but has no mechanisms to separate physiological from environmental signals. The model learns a mapping from pixels to pulse, but this mapping collapses when test conditions are different from training (e.g., different lighting or camera sensors).

Our RDG mechanism masks motion-corrupted regions using motion supervision, preventing facial expression motions from propagating through attention. DAL uses a background reference to estimate illumination changes directly, enabling the removal of non-periodic lighting variations. Orthogonality loss forces the separation of physiological and environmental signals in latent space to improve cross-domain generalization. The Adaptive Frequency Window dynamically adapts the band-pass based on measured facial activity, avoiding both low-frequency motions and exclusion of high physiological rates.

### 4.10. Research Limitations and Future Directions

#### 4.10.1. Limitations

Despite the accuracy and robustness of the TH-STT architecture, certain constraints remain that provide opportunities for further refinement:

- Compression Sensitivity: High video compression rates can degrade the signal-to-noise ratio. While the DAL head mitigates environmental noise, the loss of subtle intensity variations due to quantization in heavily compressed streams remains a challenge for sub-millimeter physiological changes. Additionally, the reaction head will suffer from detecting reactions used in weighing the face ROIs
- Extreme Pose Variations: While the RDG head effectively manages standard facial expressions and minor rotations, extreme rigid motion (e.g., head turns exceeding 45) can lead to temporary Phase Drifts as spatial tokens lose correspondence with the underlying tissue.
- Computational Complexity for Edge Deployment: The current implementation uses a ViT-Small backbone. Proposed work is efficient for workstation-class GPUs; the computational overhead and memory footprint may limit real-time execution on mobile devices or embedded systems without further optimization.
- Limitations we cannot solve: Stabilizing the camera’s position is crucial. Moving objects can be tracked, but handling an unstable camera is extremely difficult.

#### 4.10.2. Future Works

Building upon the results of this study, future research will focus on the following trajectories:

- Lightweight Architectures: We aim to explore the transition from ViT-Small to a highly optimized ViT-Tiny or MobileViT backbone. We want to use Knowledge Distillation to move the TH-STT’s rich spatio-temporal features into a lighter model with a lot fewer parameters.
- Edge-Device Optimization: Future iterations will involve the implementation of TensorRT or CoreML optimizations to take advantage of hardware acceleration on mobile Processing Units. This will allow the system to operate in real-time on standard smartphones and be viable for telemedicine applications.
- Multi-Parameter Physiological Sensing: We plan to extend the triple-head model to involve additional biometrics such as Respiratory Rate (RR) and Oxygen Saturation SpO_2_.

## 5. Conclusions

In this work, we proposed the Triple-Head Spatio-Temporal Transformer (TH-STT) to improve the robustness of rPPG in real-world scenarios. The model is designed to separate the physiological pulse signal from noise caused by facial motion and changing illumination. The framework includes Reaction-Driven Gating with Dynamic Anchor Locking to reduce motion-related distortions and compensate for lighting variations. Results across multiple benchmark datasets show that TH-STT has robust performance under challenging conditions. Multi-task learning and signal separation have proved their effect on the accuracy of rPPG systems.

## Figures and Tables

**Table 2:** Comparison of Transformer-based rPPG models in terms of noise robustness.

**Model**	**Architecture**	**Noise Robustness Mechanism**	**Robustness Type**
PhysFormer [16]	Dense Transformer	Temporal Difference (TD) features	Implicit
RhythmFormer [19]	Hierarchical periodic Transformer	Periodic Query/Refinement	Implicit
VidFormer [18]	Global Temporal Attention + CNN	Spatio-temporal redundancy	Implicit
Shao et al. [22]	Swin Transformer	Global Interference Sharing & Background Ref.	Explicit
TH-STT (Our proposed)	Sparse ST-Transformer	RDG, DAL Anchor, & AFW	Hybrid (Implicit + Explicit)

**Table 3:** Architectural Comparison and Integration Logic of multi-component models.

Architecture	Motion Handling	Illumination Handling	Post-Processing	Integration Logic
MTTS-CAN/TS-CAN [15]	Temporal Shift	Global Average	Static Bandpass (0.75–2.5)	Passive (Multi-task Loss)
PhysNet [6]	3D-Convolutions	Normalized Input	Static Bandpass	Passive (Feature-Fusion)
Shao et al. [22]	Landmark- based STMap	Global Sharing/Background	Static Bandpass	Softmax Similarity
VidFormer [18]	Dense ST-Attention	Generic Attention	Static Bandpass	End-to-End Mapping
TH-STT (Ours)	RDG (Reaction)	DAL (Anchor)	AFW (Adaptive)	Active (Closed-Loop)

**Table 4:** Different Dataset details.

Dataset	Videos	Subjects	Main Challenge	Illumination Source	Compression	Ground Truth
UBFC-rPPG	42	42	Spontaneous Reaction	Indoor	No compression (data rate~220 Mbps)	CMS50E (PPG)
VIPL-HR	2378	107	Sensor and Scale Diversity-Motion	Indoor/dark/bright	Moderate (data rate~5.17 Mbps)	BVP/ECG
COHFACE	160	40	Illumination Compression rate	Lab/Natural	High (data rate~250 kbps)	BVP

**Table 5:** Performance across benchmark datasets.

Method	UBFC		VIPL-HR		COHFACE	
	MAE (bpm) ↓	RMSE (bpm) ↓	MAE (bpm) ↓	RMSE (bpm) ↓	MAE (bpm) ↓	RMSE (bpm) ↓
CHROM [12]	4.06	8.83	11.37	16.99	3.82	6.8
POS [13]	4.08	7.62	10.8	14.8	3.14	10.57
Ts-CAN [15]	1.70	2.72	-	-	-	-
DeepPhys [14]	6.25	10.81	11.04	13.82	6.56	13.84
PhysNet [6]	2.95	3.67	10.8	14.8	5.38	10.76
PhysFormer [16]	0.52	0.71	4.97	7.79	-	-
PhysFormer++ [17]	0.51	0.69	4.88	7.62	-	-
RhythmFormer [19]	0.5	0.87	**4.51**	7.98	1.17	3.36
RS-rPPG [20]	-	-	5.98	10.5	-	-
TH-STT (Ours)	**0.43**	**0.63**	4.65	**7.23**	**1.08**	**3.15**

**Table 6:** Ablation study of TH-STT components across different datasets.

Configuration	UBFC (MAE ↓)
Baseline (Shared Backbone)	0.82
+Reaction Head (DSG)	0.53
+Illumination Head (DAL)	0.70
Full TH-STT (Without AFW)	0.437
Full TH-STT (With AFW)	0.42

**Table 7:** Performance comparison across different temporal window sizes on the VIPL-HR dataset.

Window	RMSE	FLOPs (G)
64	8.62	32.7
96	7.73	46.5
128	7.23	61.1
160	7.09	83.7

**Table 8:** Standardized Per-Frame Computational and Performance Metrics.

Method	Type	Parameters (M)	FLOPs(M) /Frame	Time /Frame (ms)	RMSE
DeepPhys	2D-CNN	3.91	744	0.231	13.8
TS-CAN	2D + shift	3.91	744	0.230	14.59
PhysNet	3D-CNN	0.78	429	0.126	14.8
PhysFormer	Transformer	7.38	293	0.141	7.79
PhysFormer++	Transformer	9.79	311	0.145	7.62
RhythmFormer	Transformer	3.25	240	0.182	7.98
TH-STT (Ours)	Transformer	14.8	476	0.219	7.23

**Table 9:** Cross-dataset validation.

Method	MAE (bpm) ↓	RMSE (bpm) ↓	Pearson (ρ) ↑
PhysFormer++	1.81	3.75	0.92
RhythmFormer	1.72	3.45	0.93
TH-STT (Ours)	1.54	3.17	0.92

## Data Availability

The original contributions presented in this study are included in the article. Further inquiries can be directed to the corresponding author.
